# Design and Evaluation of Polyox and Pluronic Controlled Gastroretentive Delivery of Troxipide

**DOI:** 10.1155/2014/804616

**Published:** 2014-11-19

**Authors:** Swati C. Jagdale, Shraddha B. Kamble, Bhanudas S. Kuchekar, Aniruddha R. Chabukswar

**Affiliations:** Department of Pharmaceutics, MAEER's Maharashtra Institute of Pharmacy, MIT Campus, Kothrud, Pune 411038, India

## Abstract

*Objective*. Objective of the present work was to develop site-specific gastroretentive drug delivery of Troxipide using polymers Pluronic F127 and Polyox 205 WSR. Troxipide is a novel gastroprotective agent with antiulcer, anti-inflammatory, and mucus secreting properties with elimination half-life of 7.4 hrs. Troxipide inhibits *H. pylori*-derived urease. It is mainly absorbed from stomach. *Methods*. 3^2^ factorial design was applied to study the effect of independent variable. Effects of concentration of polymer on dependant variables as swelling index, hardness, and % drug release were studied. Pluronic F127 and Polyox 205 WSR were used as rate controlled polymer. Sodium bicarbonate and citric acid were used as effervescent-generating agent. *Results*. From the factorial batches, it was observed that formulation F5 (19% Pluronic F127 and 80% Polyox 205 WSR) showed optimum controlled drug release (98.60% ± 1.82) for 10 hrs with ability to float >12 hrs. Optimized formulation characterized by FTIR and DSC studies confirmed no chemical interactions between drug and polymer. Gastroretention for 6 hrs for optimized formulations was confirmed by *in vivo* X-ray placebo study. *Conclusion*. Results demonstrated feasibility of Troxipide in the development of gastroretentive site-specific drug delivery.

## 1. Introduction

Oral controlled release dosage forms have been developed over the past three decades due to their considerable therapeutic advantages such as ease of administration, patient compliance, and flexibility in formulation. Gastroretentive dosage form can remain in the gastric region for several hours and hence significantly prolong the gastric residence time of drugs. Prolonged gastric retention improves bioavailability, reduces drug wastage, and improves solubility of drugs that are less soluble in a high pH environment. It is also suitable for local drug delivery to stomach and proximal small intestine. Several approaches have been attempted in the preparation of gastroretentive drug delivery system as floating, swellable and expandable, high density, bioadhesive, altered shape, gel forming solution or suspension system and sachet systems [1–3].

In case of certain drug candidate, greater therapeutic value is achieved by increasing the gastric retention time. Some examples include drugs absorbed from the proximal part of the gastrointestinal tract, for example, diltiazem; drugs that get degraded in intestinal alkaline pH, for example, bromocriptine; drugs that are absorbed primarily in the stomach, for example, albuterol; and drugs that degrade in the colon, for example, captopril. In few conditions local and preferably sustained delivery to stomach and proximal part of small intestine is desired, for example, proton pump inhibitors for ulcers. Prolonged retention of drug may also increase bioavailability and therapeutic efficacy of the drug. The dose size may also be reduced in some cases for gastroretention system [4–6].

*Helicobacter pylori (H. pylori)* were the first isolated microaerophilic gram-negative bacteria from the gastric mucosa of gastritis patients by Marshall and Warren in the 1980s.* Helicobacter pylori* have become recognized as a major gastric pathogen with worldwide distribution.* H. pylori*, a prevalent human-specific pathogen, are a causative agent in chronic active gastritis, gastric and duodenal ulcers, and gastric adenocarcinoma, one of the most common forms of cancer in humans. The poor patient compliance to antibiotic because of high risk of resistance gives rise to the need of new medicines with better effectiveness and simpler regimens. Eradication of* H. pylori* is difficult because of the organism's habitat in the stomach under the mucus layer [7–11].

A logical way to improve the effectiveness of therapy is to develop a drug delivery system which can reside in the stomach for longer duration and release drug as long as possible in the ecological niche of the bacterium. Gastroretentive drug delivery system (GRDDS) is an ultimate solution for this [12].

Troxipide is a novel gastroprotective agent with antiulcer, anti-inflammatory, and mucus secreting properties. It is designated chemically as 3,4,5-trimethoxy-N-(3-piperidyl) benzamide. Troxipide has cytoprotective properties on the gastric mucosa. It is used in amelioration of gastric mucosal lesions (erosion, hemorrhage, redness, and edema) in the acute gastritis, acute exacerbation stage of chronic gastritis. Troxipide inhibits* H. pylori*-derived urease, a multimeric nickel-containing enzyme that catalyses the hydrolysis of urea to yield ammonia and carbonic acid, which damage host tissues and trigger inflammatory response, including recruitment of leukocytes and triggering of the oxidative burst in neutrophils. Troxipide has relative bioavailability of 99.6%. The dose of drug is 100 mg thrice a day. The half-life of drug is about 7.4 hrs. The drug is soluble only in acidic pH and is mainly absorbed from stomach. Due to this reason Troxipide was selected for present work aiming at retention of drug in stomach to increase site-specific absorption of Troxipide by developing gastroretentive drug delivery system to stomach [13, 14].

## 2. Materials and Methods

### 2.1. Materials

Troxipide was obtained as gift sample from Chiral Bio-Life Sciences, Hyderabad. Polyox 205WSR was gifted by Colorcon Asia Pvt. Ltd., Mumbai. Pluronic F127 was purchased from Analab Fine Chemicals, Mumbai. All other chemicals used were of analytical grade.

Polyethylene oxide (Polyox) is nonionic, highly swelling, thermoplastic, and water soluble resin. Polyox 205 WSR has molecular weight of 600,000 with viscosity of 4500–8800 cPs (5% solution). Upon exposure to water or gastric juices, it hydrates and swells rapidly to form hydrogels with properties ideally suited for controlled drug delivery vehicles. It has successful application in controlled release solid dose matrix systems, transdermal drug delivery, and mucosal bioadhesive. Pluronic F-127 is a nonionic, surfactant polyol (molecular weight approximately 12,500 daltons). Pluronic F-127 (Poloxamer 407, PF-127) forms a thermoreversible gel. This characteristic has allowed PF-127 to be used as a carrier for most routes of administration including oral, topical, intranasal, vaginal, rectal, ocular, and parenteral routes.

### 2.2. Preparation of Gastroretentive Floating Tablets (GRFTs)

Floating tablets were prepared by direct compression technique. Dose of Troxipide was fixed to 100 mg. Each powder (Troxipide, sodium bicarbonate, citric acid, dicalcium phosphate, Polyox 205 WSR, and Pluronic F127) was passed through 60 mesh sieve (Retch). Powder mixing was carried out in polythene bag for 15 minutes. Powder blend was lubricated with talc and magnesium stearate. Mixing was continued for another 10 min; tablets were compressed using 8-station rotary press tablet compression machine (Cadmach, Rimek Minipress). Trial batches were prepared to optimise the concentration of sodium bicarbonate and polymers. Concentration of sodium bicarbonate (16%) and citric acid (2%) was finalised. Based on the results of trial batches, tablets were prepared using 3^2^ factorial design. 3^2^ factorial design was applied to establish the interrelationship between the selected variables. The design includes 2 factors evaluated at 3 levels each. The factorial design contains 9 experiments. In this design 3 levels of concentrations are used having lowest, middle, and highest concentrations of each variable and coded as −1, 0, and +1, respectively. The coded levels and the exact concentration of the variables used in different formulations are shown in Table 1. Two variables were used and factorial is run as per the sequence shown in Table 2. In the present study, independent variables were concentration of Pluronic F127 (*X*_1_) and Polyox 205 WSR (*X*_2_). Three concentrations were decided such that the difference between two consecutive levels is the same. The dependent variables were *Z*_1_—swelling index, *Z*_2_—hardness, *Z*_3_—% drug release, and *Z*_4_—floating time as shown in Table 1. Nine batches were formulated as per the factorial design as shown in Table 2.

### 2.3. Evaluation of Powder Blend

Powder blend was evaluated for precompression parameters as angle of repose, bulk density, tapped density, Carr's index, and % compressibility index [15].

### 2.4. Evaluation of GRFTs

GRFTs were evaluated for hardness (Monsanto hardness tester), friability (Roche friabilator), weight variation, % drug content, swelling index,* in vitro* buoyancy, and* in vitro* drug release. The results are expressed as mean ± SD (*n* = 3).

### 2.5. Uniformity of Content

Tablet powder was added to 10 mL of 0.1 N HCl and drug solution was filtered through Whatman paper. The sample was analyzed for drug content by UV spectrophotometer (Varian Cary 100) at 258 nm.

### 2.6. Tablet Floating Behaviour

Floating time was determined using USP dissolution apparatus-II in 900 mL of 0.1 N HC1 at 37 ± 0.5°C. The duration for floating (floating time) was the time the tablet remains afloat in the dissolution medium [16].

### 2.7. Swelling Index (SI)

SI of all factorial batches was calculated by using USP dissolution apparatus type I. In this study six tablets were placed in basket of dissolution apparatus with 0.1 N HCl as dissolution medium at 37 ± 0.5°C. Tablets were withdrawn at a time interval of 60 min, blotted with tissue paper to remove the excess water, and weighed on the analytical balance (Shimadzu, AUW220D). The study was conducted in triplicate [17, 18]. Swelling index was calculated as

(1) Swelling  index  (SI)=W1−W0W0×100,

where *W*_*t*_ is weight of tablet at time *t* and *W*_0_ is initial weight of tablet.

### 2.8. *In Vitro* Dissolution Study

All factorial batches were studied for* in vitro* drug release analysis. The dissolution test was performed using 900 mL of 0.1 N HCL, at 37 ± 0.5°C and 50 rpm speed (USP dissolution apparatus type II). Aliquots of dissolution medium were withdrawn at 1 hr time interval up to 10 hours. Aliquots were filtered and content of Troxipide was determined using UV spectrophotometer at 258 nm. Dissolution studies were performed in triplicate.

### 2.9. *In Vivo* X-Ray Placebo Study

X-ray technique was used to determine the gastric residence time of the tablets.* In vivo* X-ray placebo study was carried out by administering formulation (F5) which was prepared by replacing drug (100 mg) with barium sulphate. Three healthy volunteers of mean age 25 ± 2 yrs and mean weight 60 ± 10 Kg were selected for study. The written consent of the human volunteers was taken before participation and the studies were carried under the supervision of an expert radiologist and physician. The prepared tablet was administered to every subject in fed state. Gastric radiography was carried out at 0.5, 2, 4, and 6 hrs. All work was conducted in accordance with the Declaration of Helsinki [18].

### 2.10. Kinetic Modelling of Drug Release Profiles

The dissolution profile of all the batches was fitted to the following models:

(2) Zero-order  (F=k×t)First-order  (ln⁡  F=k×t)Hixson-Crowell  (F=1001−1−kt3)Korsmeyer-PeppasF=ktnHiguchimatrixF=k√t,

where *F* is the fraction of drug release, *k* is the release constant, *t* is the time, and *n* is diffusional coefficient [19].

### 2.11. Statistical Analysis of Drug Release Profiles

1. Model fitting was carried out using PCP DISSO v2.08 software.
2. Similarity factor was calculated by comparing dissolution profile of formulation with marketed formulation using BIT software.
3. The factorial data were analyzed using design expert 8.0.7.1 version software.

### 2.12. Stability Study

Optimized formulation (F5) was sealed in aluminium packaging coated inside with polyethylene. This was kept in the humidity chamber at 40°C and 75% RH and sampling was carried out for 1, 2, and 3 months (Thermo-Lab). Samples were analyzed for the physical appearance, floating properties, drug content, and drug release study [20, 21].

## 3. Results and Discussion

### 3.1. Preliminary Trial Batches

Formulations containing 20% and 10% of sodium bicarbonate alone did not show any floating, whereas formulation containing 16% sodium bicarbonate along with citric acid (2%) showed floating. Further trial batches were conducted using Pluronic F127 and Polyox WSR 205 keeping concentration of gas forming agent constant. It was observed that formulation containing 120 mg Polyox WSR 205 showed immediate floating, but the formulations dissolved within 3 hours, whereas 100 mg pluronic F127 alone did not show floating. Formulations containing combination of Polyox WSR 205 and Pluronic F127 showed floating within 5 min with sustained drug release for more than 10 hours. Hence combinations of these two polymers were used to get controlled drug release.

### 3.2. Characterization of Drug and Excipients

*UV Spectroscopy (Determination of λ* *Max)*. The drug obeys Beer-Lambert's law in the range 2–10 *μ*g/mL. All other analytical parameters as precision, accuracy, and robustness showed values of standard deviation and relative standard deviation within limit (not more than 2). LOD and LOQ found were 0.451 *μ*g/mL and 1.408 *μ*g/mL, respectively.

### 3.3. Drug Excipient Compatibility Study

#### 3.3.1. Infrared (IR) Spectroscopic Study

**Figure 1:**
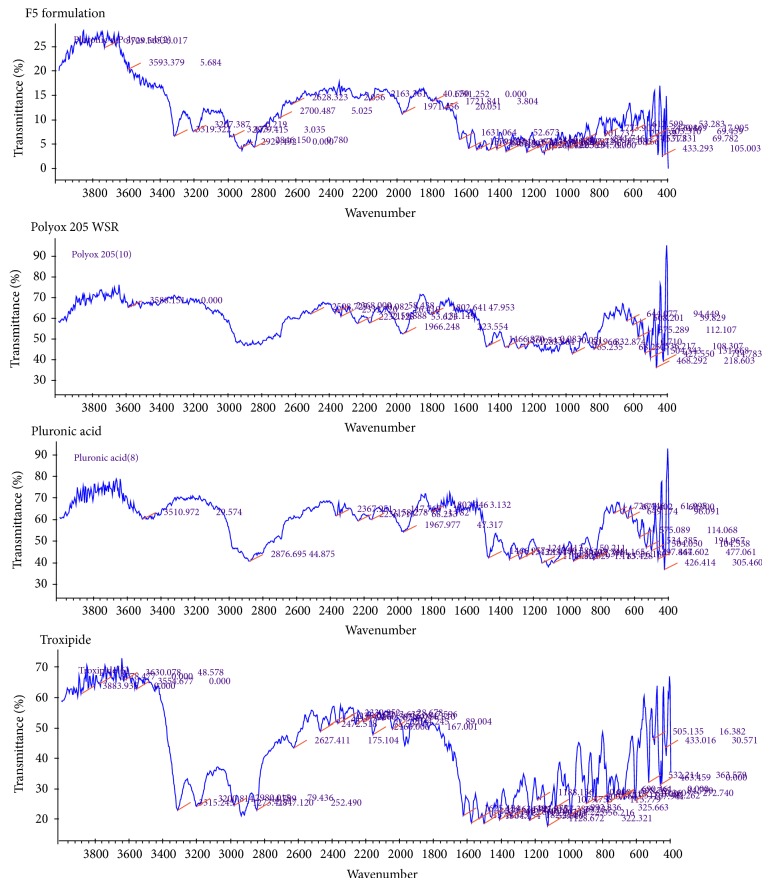
IR spectroscopic study of drug, polymer, and formulation (F5).

Figure 1 shows infrared spectroscopic scan of Troxipide, polymer, and formulation. Characteristics peaks of Troxipide were found in the range for as 3500–3300 cm^−1^, N–H stretch, C–H stretch aromatic at 2960–2850 cm^−1^, and c=0 stretch at 1680–1630 cm^−1^ [20]. IR data indicated that there was no chemical interaction between Troxipide and excipients as there were no changes in the characteristic peaks of Troxipide in the IR spectra of mixture of drug and excipients as compared to IR spectra of pure drug.

#### 3.3.2. Differential Scanning Calorimetry (DSC) Study

**Figure 2:**
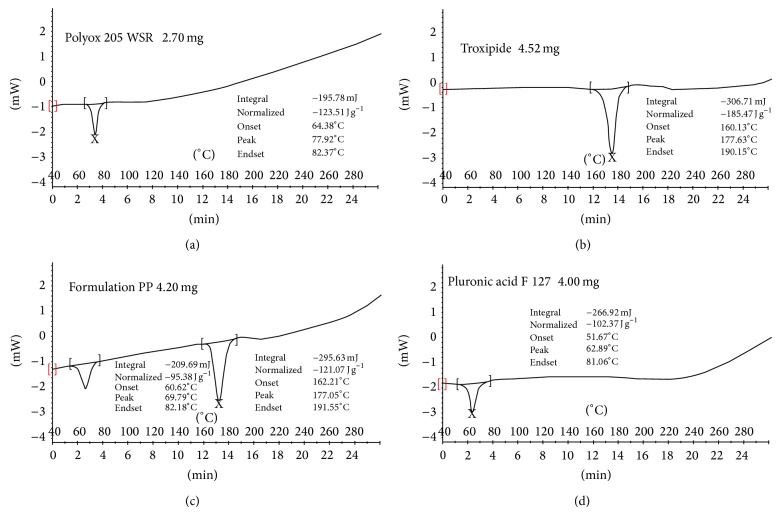
DSC thermograph of (a) Polyox 205 WSR, (b) Troxipide, (c) F5 formulation, and (d) Pluronic F127.

DSC (Figure 2) showed melting point of Troxipide, Polyox WSR 205 and Pluronic F127 in the range of 177–181°C, 75–80°C, and 54–56°C, respectively. Thermographs obtained by DSC studies revealed that there is no significant difference in the melting point of the drug in the thermographs of drug and formulation. It was concluded that the drug is in the same pure state even in the formulation without interacting with the polymers.

### 3.4. Evaluation of Powder Blend (F1–F9)

Precompression parameters for powder blend showed results within specified limits. Results were expressed in the range for angle of repose (23–27°C), bulk and tapped density (0.40–0.53 g/cm^2^), and % compressibility index (10–12%), and Carr's index was 8 to 11. These values indicated that all the powder blends showed good flow property [22].

### 3.5. Evaluation of Tablets

All postcompression parameters for batch (F1–F9) are as shown in Table 3 which lies within specified limits in IP [23].

### 3.6. Swelling Index

**Figure 3:**
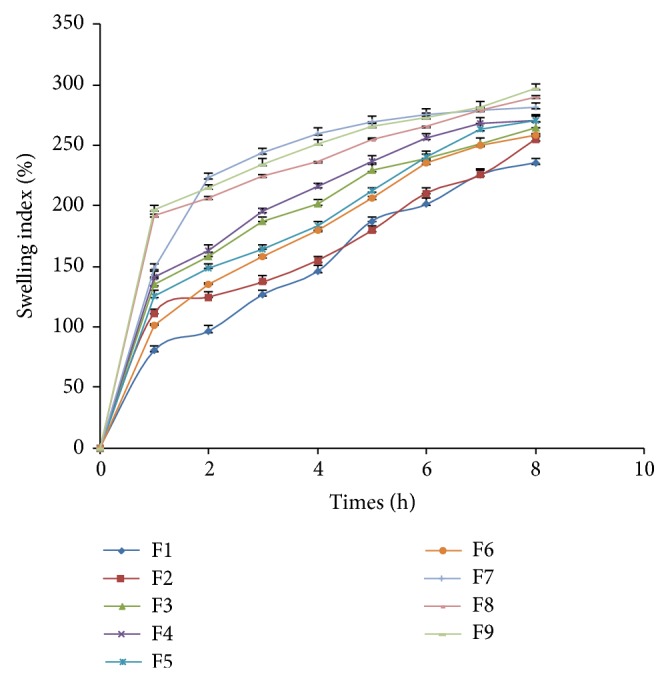
Swelling indices profile of factorial batches F1–F9.

Swelling study was performed on all the batches (F1-F2) for 8 hrs and the results of swelling index are given in Figure 3. Swelling is a vital factor to ensure floating and drug release. In GRFT, drug is dispersed throughout the polymer. When GRFTs are brought into contact with dissolution medium, polyether chains of Polyox 205 WSR formed hydrogen bonds with water and polymer tends to hydrate forming a superficial gel which eventually erodes as the polymer dissolves. Same time pluronic swelled in water and form a rapid gel layer that impeded erosion of polymers and because of that entrapped drug release at predictable rate. From the swelling index study of all the batches, it was observed that increase in the concentration of polymers increases the swelling property of the tablets. Similar results have been obtained by using xanthan combined with HPMC due to quick hydration and subsequent gel formation in case of terbutaline sulfate matrix tablets [24]. In other cases of floating delivery of simvastatin with Polyox WSR N12k and HPMC K4M, it was found that Polyox WSR N12K has more effect on swelling as compared to HPMC K4M [25].

### 3.7. *In Vitro* Dissolution Study

**Figure 4:**
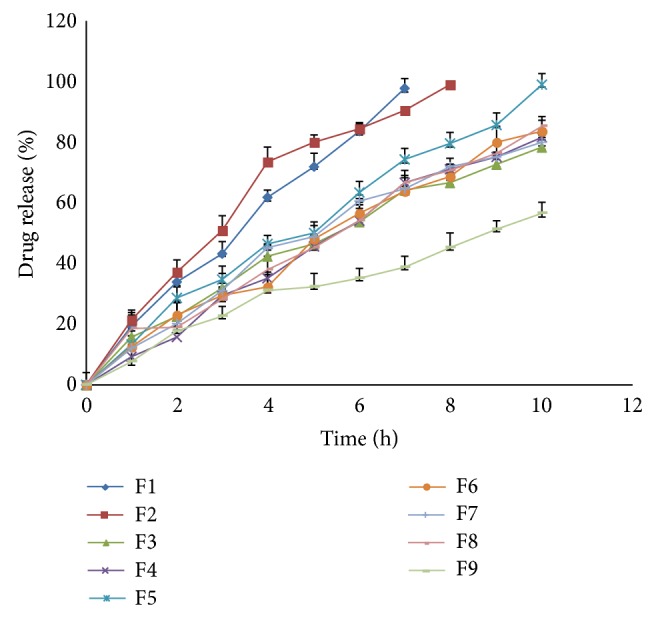
Dissolution profile of factorial batches F1–F9 of Pluronic F127 and Polyox 205 WSR.

Percent drug release after 10 hours is as shown in Figure 4. The drug release profile of formulations F1–F9 indicated that as the concentration of polymers increases, the drug release was retarded. After comparing release profile of all the batches, it was observed that the formulations containing high concentration of polymers had shown retardation of drug release to greater extent. Batches F1, F2, and F9 fail to comply with standards for drug release as mentioned for modified release tablet in USP29.

### 3.8. Mathematical Modelling and Drug Release Kinetics

Drug dissolution from solid dosage forms has been described by kinetic models in which the dissolved amount of drug is a function of the test time. The dissolution data for GRFT (F1–F9) was fitted to various drug release kinetic models. Based on the *R*-value, the best fit model was selected as shown in Table 4. From* in vitro* dissolution studies and the response surface curves, it was observed that the drug release pattern was influenced by the variation in the polymers concentration. F1, F2, F4, F5, F6, and F9 follow Peppas type of release pattern; this indicates that the release mechanism is not well known or more than one type of release phenomena is involved as Fickian diffusion (Higuchi matrix), anomalous transport, and zero-order release. An “*n*” value 0.5 is considered consistent with diffusion controlled release, whereas a value of 1.0 indicates a zero-order release behaviour and intermediate value (0.5 > 1.0) is defined as anomalous nonfriction transport mechanism. F7 showed Hixson-Crowell as best fit model. Hixson-Crowell model applies to pharmaceutical dosage form as tablet where the dissolution occurs in the planes that are parallel to drug surface if the tablet dimensions diminish proportionally in such a manner that the initial geometric forms keep constant all the time. When this model is used it is assumed that the release rate is limited by the drug particles dissolution rate and not by the diffusion that might occur through the polymer matrix. F3 and F8 showed zero order as best fit model indicated that the combination effects of diffusion and polymer relaxation play a role in drug release. From the* in vitro* dissolution studies and response surface curves, it was observed that the drug release pattern was affected by polymer concentration.

Polyether chains of Polyox 205 WSR are forming a superficial gel which erodes as the polymer dissolves and at the same time pluronic swelled and formed a rapid gel layer. Due to this as the concentration of both is varied in different proportions all batches have shown different release kinetics data. In one of the studies it was found that synthetic polymer showed less mass loss and water uptake compared to natural gums and hydration rate of this cellulosic polymer relates to its hydroxypropyl substitutes percentage in HPMC-K4M and gave Peppas model with non-Fickian diffusion in case of terbutaline [24]. In another study for levofloxacin tablet, it was found that higher initial drug dissolution was observed in tablets containing higher proposition of HPMC compared to Gelucire [26]. In study of simvastatin formulations lower concentration of HPMC K4M and Polyox showed maximum drug release in 10 hours indicating that both polymers have significant release retardant effect [25].

### 3.9. Response Surface Plots: [27–29]

**Figure 5:**
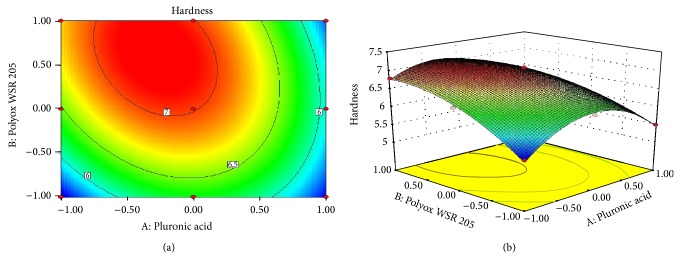
(a) Contour plot showing the relationship between various levels of Pluronic F127 and Polyox 205 WSR and (b) response surface plot showing the influence of Pluronic acid F127 and Polyox 205 WSR on hardness.

**Figure 6:**
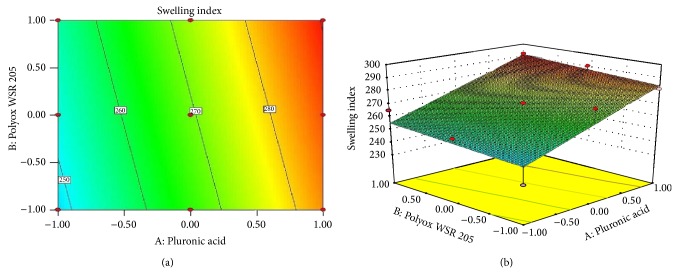
(a) Contour plot showing the relationship between various levels of Pluronic F127 and Polyox 205 WSR and (b) response surface plot showing the influence of Pluronic acid F127 and Polyox 205 WSR on SI.

Response surface methodology (RSM) is a collection of mathematical and statistical techniques for empirical model building. RSM was used to determine the effect of independent variables on all possible dependent variables. 3^2^ full factorial design was selected to study the effect of independent variables Pluronic F127 and Polyox 205 WSR on dependent variables hardness and swelling index (Figures 5 and 6 and Table 5). A statistical model incorporating interactive and polynomial terms was utilized to evaluate the responses:

(3) Hardness=7.01−0.28A+0.37B−0.30AB−0.82A2−0.37B2,

(4) Swelling  index=269.28+17.74A+3.40B,

where *A* and *B* represent the effect of variables, that is, concentration of Pluronic F127 and Polyox 205 WSR, respectively.

Statistical optimization which was carried out by the software suggested that quadratic model was followed for first response and linear model was followed for second response. The *P* value of hardness and swelling index was 0.0022 and 0.0139, respectively. The *P* value was less than 0.0500 which indicated that the model was highly significant. This receives confirmation from the mathematical model (ANOVA) generated for responses indicated in Table 5. From (3) it was observed that Polyox 205 WSR has significant effect on hardness as compared to Pluronic. Low value of *AB* coefficient also suggested that the interaction between *A* and *B* has not shown a significant effect on hardness. From (4) it was observed that both polymers have significant effect on swelling index. High level of *A* and low level of *B* were responsible for swelling index. The response surface plots and contour plots showing the effect of polymer on hardness and swelling index are as shown in Figures 5 and 6, respectively.

### 3.10. Validation of Statistical Model

After statistical analysis by design expert software, the experimental values were found to be very close to the predicted values and, hence, the model was successfully validated.

### 3.11. Similarity Factor Study

Troxipide (Troxip 100 mg; Zuventus Healthcare Ltd.) was compared with optimized formulation F5. Similarity factor *f*_2_ obtained was 8 which is less than the 50. This confirms no similarity in the drug release of both test and marketed formulation because marketed formulation was immediate release while the optimized formulation was sustained release.

### 3.12. *In Vivo* Placebo X-Ray Study

**Figure 7:**
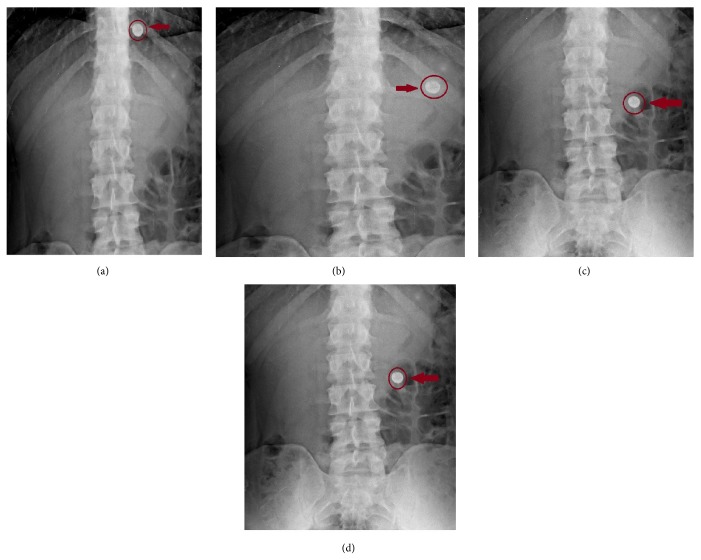
X-ray of formulation F5 after time interval: (a) 0.5 hrs; (b) 2 hrs; (c) 4 hrs; and (d) 6 hrs.

*In vivo* evaluation was carried out in fed state. The behavior of tablet was studied in volunteers using radiographic imaging technique. After administration of F5 optimised formulation (containing barium sulphate) the radiographs were taken after 0.5, 2, 4, and 6 hrs. The radiographs taken after 0.5 hrs (Figure 7(a)) imply the buoyancy of the tablets. Next radiograph, taken at 2 hrs (Figure 7(b)), shows change in position of tablet; this showed that tablet did not adhere to gastric mucosa. Figures 7(c) and 7(d) showed the positions of tablet in stomach after 4 and 6 hours, respectively. X-ray study indicated no adherence of tablet to gastric mucosa. It was observed that the tablets remained afloat in stomach till the time period of 6 hrs giving successful gastroretention property. This indicated that successful targeting of drug can take place in stomach.

### 3.13. Stability Study

Formulation is found to be stable since there were no significant change in the physical appearance, floating properties, drug content, and drug release studies. It can be concluded that the floating tablets of Troxipide F5 were physically as well as chemically stable under these storage conditions for at least 3 months.

## 4. Conclusion

Recently it was revealed that antibiotics used for the treatment of* H. pylori* infections showed the poor patient compliance because of high risk of resistance. As the pathogen shows its habitat in the stomach, the site-specific drug delivery with prolong gastric residence is needed. Gastroretentive drug delivery system of Troxipide was successfully prepared by using 3^2^ factorial design. Optimized batch F5 showed gastric residence time more than 10 hrs with maximum drug release.* In vivo* evaluation by X-ray technique confirmed that tablet remained in the stomach for 6 hrs. Therefore optimized formulation F5 may become a logical way to improve the effectiveness of site-specific therapy against* H. pylori* infections with cytoprotective property on the gastric mucosa. However, there is further need of investigation for clinical acceptance of this novel drug delivery system.

## Figures and Tables

**Table 1:** Variables and coded levels data.

Variables used	Coded levels		
	−1	0	1
Pluronic F127	25.2	35	43.2
Polyox 205 WSR	135	145	153

**Table 2:** Formulation of gastroretentive floating tablet.

Batch code	Coded level		Pluronic F127 (mg)	Polyox WSR 205 (mg)	Sodium bicarbonate (mg)	Citric acid (mg)	Magnesium stearate (mg)	Talc (mg)	Di calcium phosphate (mg)
	Variable 1 (Pluronic F127)	Variable 2 (Polyox WSR 205)							
F1	−	−	25.2	135	58	10	1	1	36
F2	−	0	25.2	145	58	10	1	1	26
F3	−	+	25.2	153	58	10	1	1	18
F4	0	−	35	135	58	10	1	1	26
F5	0	0	35	145	58	10	1	1	16
F6	0	+	35	153	58	10	1	1	8
F7	+	−	43.2	135	58	10	1	1	18
F8	+	0	43.2	145	58	10	1	1	8
F9	+	+	43.2	153	58	10	1	1	0

Weight is expressed as mg per tablet. Total weight of tablet was 370 mg.

**Table 3:** Physical characteristics of GRFTs (F1–F9).

Batch code	Tablet weight (mg) *N* = 6	Hardness (kg/cm^2^) *N* = 6	Drug content (%) *N* = 10	Tablet friability (%) *N* = 10	Floating lag time (s) *N* = 6	Total floating duration (h) *N* = 6
F1	369.32 ± 2.70	5.5 ± 0.32	98.36	0.456 ± 0.03	39	>12
F2	372.60 ± 1.50	6.4 ± 0.51	99.56	0.543 ± 0.01	52	>12
F3	368.06 ± 2.33	6.8 ± 0.65	101.60	0.445 ± 0.04	64	>12
F4	368.68 ± 2.15	6.2 ± 0.31	98.60	0.413 ± 0.02	59	>12
F5	369.85 ± 1.75	7.1 ± 0.58	100.01	0.555 ± 0.01	60	>12
F6	371.20 ± 2.94	7 ± 0.73	99.50	0.658 ± 0.02	74	>12
F7	370.99 ± 1.75	5.5 ± 0.68	97.62	0.520 ± 0.03	61	>12
F8	372.33 ± 2.56	5.9 ± 0.55	102.08	0.644 ± 0.01	56	>12
F9	368.20 ± 2.11	5.6 ± 0.51	97.20	0.689 ± 0.04	76	>12

**Table 4:** Mathematical modeling and release kinetics for formulations F1–F9.

Batch code	Zero order	First order	Matrix	Hixson-Crowell	Korsmeyer-Peppas		Best fit model
	(*R* ^2^)	(*R* ^2^)	(*R* ^2^)	(*R* ^2^)	(*R* ^2^)	(*n*)	
F1	0.9941	0.8793	0.9618	0.9587	0.9995	0.8301	Korsmeyer-Peppas
F2	0.9748	0.9328	0.9678	0.9916	0.9870	0.8733	Korsmeyer-Peppas
F3	0.9801	0.9382	0.9478	0.9654	0.9789	0.6896	Zero order
F4	0.9937	0.9787	0.9396	0.9913	0.9953	0.9863	Korsmeyer-Peppas
F5	0.9912	0.8840	0.9574	0.9626	0.9937	0.8729	Korsmeyer-Peppas
F6	0.9950	0.9640	0.9426	0.9855	0.9959	0.8997	Korsmeyer-Peppas
F7	0.9711	0.9946	0.9696	0.9967	0.9841	0.7596	Hixson-Crowell
F8	0.9908	0.9657	0.9397	0.9849	0.9644	0.8007	Zero order
F9	0.9541	0.9839	0.9796	0.9778	0.9885	0.6185	Korsmeyer-Peppas

*n*: diffusional exponent.

**Table 5:** ANOVA used to generate statistical models.

Response model	Sum of squares	Df	Mean square	*F* value	*P* value	SD	*R* ^ 2^	Adequate precision
Hardness	3.25	5	0.65	79.80	0.0022	0.090	0.9925	0.9801
Swelling index	1957.54	2	978.77	9.48	0.0139	10.16	0.7596	0.6795
